# Pro-tobacco marketing and anti-tobacco campaigns aimed at vulnerable populations: A review of the literature

**DOI:** 10.18332/tid/111397

**Published:** 2019-09-18

**Authors:** Tess Boley Cruz, Shyanika W. Rose, Brianna A. Lienemann, M. Justin Byron, Helen I. Meissner, Lourdes Baezconde-Garbanati, Li-Ling Huang, Dana M. Carroll, Claradina Soto, Jennifer B. Unger

**Affiliations:** 1 Keck School of Medicine, University of Southern California, Los Angeles, United States; 2 Truth Initiative Schroeder Institute, Washington, United States; 3 Center for Health Equity Transformation and Behavioral Science, University of Kentucky College of Medicine, Lexington, United States; 4 Moores Cancer Center, University of California San Diego, San Diego, United States; 5 Department of Family Medicine, School of Medicine, University of North Carolina at Chapel Hill, Chapel Hill, United States; 6 Office of Disease Prevention, National Institutes of Health, Bethesda, United States; 7 Global Health and Health Security, Taipei Medical University, Taipei, Taiwan; 8 Masonic Cancer Center, University of Minnesota, Minneapolis, United States

**Keywords:** pro-tobacco, anti-tobacco, marketing, campaigns, vulnerable populations

## Abstract

**INTRODUCTION:**

We reviewed research literature on pro-tobacco marketing and anti-tobacco campaigns targeting eight vulnerable populations to determine key findings and research gaps. Results can inform tobacco policy and control efforts and the design of public education campaigns for these groups.

**METHODS:**

Five journal databases in medicine, communication, and science, were used to identify 8875 peer-reviewed, original articles in English, published in the period 2004–2018. There were 144 articles that met inclusion criteria on pro-tobacco marketing or anti-tobacco campaigns aimed at eight US groups: women of reproductive age, racial/ethnic minority groups (African American, Hispanic/Latino, Asian/Pacific Islander and American Indian/Alaska Native), Lesbian/Gay/Bisexual/Transgender (LGBT) populations, groups with low socioeconomic status, rural/inner city residents, military/veterans, and people with mental health or medical co-morbidities. We summarized the number of articles for each population, type of tobacco, and pro-tobacco or anti-tobacco focus. Narrative summaries were organized by population and by pro-tobacco or anti-tobacco focus, with key strategies and gaps by group.

**RESULTS:**

There were more studies on pro-tobacco marketing rather than anti-tobacco campaigns, and on cigarettes rather than other tobacco products. Major gaps included studies on Asian Americans, American Indian/Alaska Natives, pregnant women, LGBT populations, and those with mental health or medical co-morbidities. Gaps related to tobacco products were found for hookah, snus, and pipe/roll-your-own tobacco in the pro-tobacco studies, and for all products except cigarettes in anti-tobacco studies. Common tobacco industry methods used were tailoring of product and package design and messages that were used to reach and appeal to different sociodemographic groups. Studies varied by research design making it difficult to compare results.

**CONCLUSIONS:**

We found major research gaps for specific groups and tobacco products. Public education campaigns need a stronger foundation in empirical studies focused on these populations. Research and practice would benefit from studies that permit comparisons across studies.

## INTRODUCTION

Pro-tobacco marketing and anti-tobacco mass media campaigns can influence the likelihood of initiating and using tobacco^1^. While smoking has declined over the past five decades in the United States, disparities persist due to targeting by the tobacco industry and limited reach by tobacco control efforts in certain populations^2^. Analysis of both pro-tobacco marketing and anti-tobacco campaign strategies and gaps can be used to improve the reach and cultural appropriateness of public education campaigns to reduce tobacco use^3,4^.

The current study identified and analyzed research on pro-tobacco marketing and anti-tobacco mass media campaigns targeted at eight vulnerable populations in the US. These groups were considered vulnerable because of high rates of tobacco use or complications related to use. Vulnerable populations included: women of reproductive age; racial/ethnic minority groups such as African American (AA), Hispanic/Latino (HL), Asian/Pacific Islander (API), and American Indian/Alaska Native (AI/AN); Lesbian/Gay/Bisexual/and/or Transgender (LGBT) populations; people of low socioeconomic status (SES); populations in rural or inner city geographical location; military/veterans; and people with mental health or with medical comorbidities. These groups experience disparities in tobacco use, tobacco-related diseases, and/or difficulty quitting^2,5-7^. This article reviewed research on the extent, strategies and effects of pro-tobacco marketing or anti-tobacco campaigns that targeted these populations. It adds to existing literature by identifying areas of concentration and gaps in these campaigns. The results can inform prevention and cessation campaigns and tobacco control policies for at-risk populations to reduce tobacco-related disparities.

## METHODS

We conducted a review of the scientific literature to determine patterns in pro-tobacco marketing and anti-tobacco mass media campaigns targeting vulnerable populations. Citation databases included PubMed, Web of Science, ABI/Inform, Communication Source, and PsycINFO, using search terms that combined tobacco products AND marketing in the title, abstract or keywords, as shown in Table 1. Search terms encompassed all types of tobacco current at the time (e.g. chew tobacco is captured under the header ‘tobacco’). Results were limited to peer-reviewed articles in English, from 1 January 2004 through March 2018, encompassing five years before and eight years after the US enacted federal regulation of tobacco (the Family Smoking Prevention and Tobacco Control Act of 2009), yielding 8877 articles. Articles were included if they addressed one of eight vulnerable populations and pro-tobacco marketing or anti-tobacco campaigns, and were primary research studies or analysis of tobacco industry documents focused on the US. These eight groups included racial/ethnic minority groups, women who could become pregnant or have difficulty quitting while pregnant, low-income populations, sexual minorities, rural or inner city urban populations, military or veterans, or populations with either medical or mental health co-morbidities^2,5-7^. This review excluded youth and young adults as a vulnerable population separate from the other vulnerable groups, because previous reviews have focused on marketing to these populations and because the large number of studies (n=260) required a separate analysis to permit a full discussion of the work^1,2,8^.

**Table 1:** Search terms*

*Tobacco product identifier(s)*		*Marketing, advertising or social marketing identifier(s)*		*United States identifier(s)*
Cigarette; smoking; e-cigarette; e-cig; electronic cigarette; tobacco; electronic nicotine delivery system; smoking; smoke; smoker; vape; vaping; vaper; smokeless tobacco; snus; cigar; cigarillo; kreteks; bidi; filtered little cigar; little cigar; waterpipe; hookah; water pipe; narghile; arghila; dissolvable tobacco; pipe smoking; tobacco industry	AND	Social marketing; business communications; advertising; public service advertising; advertisements; advertising campaigns; mass communications communication research; propaganda; mass media; television advertising; TV advertising; radio advertising; magazine advertising; point-of-sale advertising; internet advertising; billboard advertising; social marketing; promotion; communication; media campaign; mass media; public education health education; anti-smoking; anti-tobacco; counter-marketing; counter-advertising; PSA; truth campaign; tips from a former smoker; the real cost.	AND	United States; USA; America

* Search terms were selected to find both general and specific types of tobacco and marketing/campaigns.

Major subject headings for the databases, such as ‘tobacco’, indexed many types of topics in addition to the specific terms listed.

**Figure 1:**
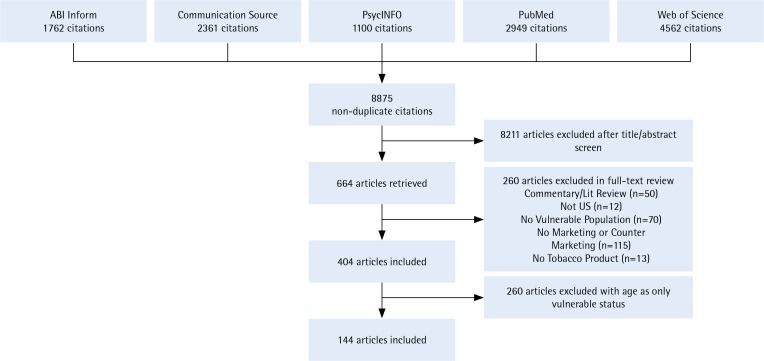
PRISMA diagram showing the flow of studies through the screening process

Two coders independently reviewed each article title and abstract, retained the articles that met the inclusion criteria, and coded their focus on pro-tobacco or anti-tobacco, vulnerable population, and type of tobacco product. Discrepant codes were reconciled by discussion, and if still unresolved, then by reading the full article. This process resulted in 146 articles included in the review (Figure 1).

### Pro-tobacco marketing or anti-tobacco campaigns

Tobacco marketing is a broad term that includes paid advertising, promotions, sponsorship, loyalty programs, product design, pricing, and more, run by tobacco manufacturers and distributors. We identified studies as pro-tobacco marketing if they analyzed planned efforts to increase tobacco use, such as greater density of tobacco billboards or promotions in retail outlets in predominantly African American communities. We also included analysis of tobacco industry documents regarding marketing plans aimed at specific groups. Anti-tobacco campaigns included public education campaigns to reduce tobacco use such as a media campaign to encourage pregnant women to stop smoking. Articles about planned media campaigns through media channels (e.g. billboards, Internet), planned media environments (e.g. stores), or movies with smoking were included, if pro-tobacco marketing or anti-tobacco campaigning was their primary focus (e.g. a main unit of analysis, predictor or outcome, not a covariate or one component of a multi-component intervention) and if they also focused on one of our eight vulnerable populations.

### Vulnerable populations

Articles were excluded if they did not focus on at least one of eight vulnerable population groups: 1) racial/ethnic minorities (AA, HL, API, AI/AN); 2) low SES; 3) pregnant women or women of reproductive age; 4) populations in inner city urban or rural areas; 5) military or veterans; 6) sexual minorities (LGBT); 7) people with mental health disorders; and/or 8) people with medical comorbidities. Studies were included if the population was an intended part of the sample in sufficient numbers to draw conclusions about that population, or the only population in the study; and excluded if the vulnerable population variable was a covariate of another analysis or the focus was on a non-vulnerable group (e.g. men as a general group).

### Tobacco products

Included articles referenced one or more tobacco products: cigarettes, cigars/little cigars/cigarillos; smokeless tobacco (ST) including chew, spit or snus; hookah/waterpipe, electronic cigarettes/vaping, pipe or roll-your-own tobacco; a specific brand or type of product (e.g. menthol cigarettes); or ‘tobacco’ as a non-defined general category.

Due to the wide variation in measurement and broad scope of the review, results were summarized narratively regarding marketing practices and outcomes.

## RESULTS

Of the 144 included articles, 64% (n=93) were considered pro-tobacco, which means they focused on tobacco advertising, promotional efforts and/or placement in films and television. There were fewer studies (38%, n=55) on anti-tobacco efforts, which means they analyzed planned prevention and cessation campaigns. Two of the articles were considered both pro-tobacco and anti-tobacco because they analyzed both perspectives (so percentages exceed 100%). Most (77%, n=111) focused on cigarettes, 17% (n=25) on ‘tobacco’ as a general product category, 5% (n=8) on e-cigarettes and 5% (n=8) on cigars, as shown in Table 2. One article focused on hookah/waterpipe, and none on pipe or roll-your-own tobacco.

**Table 2:** Articles by tobacco focus and type of product

*Product*	*Pro-tobacco marketing*	*Anti-tobacco campaigns*
Cigarettes	57^a^	55^b^
Cigars	8c	0
Smokeless	7^d^	0
Pipe/RYO	0	0
E-cigarettes	8^e^	0
Snus	1^f^	0
Hookah	1^g^	0
Other	3^h^	2^i^
General tobacco	22^j^	3^k^
Total	93	55
Total articles N=144. Two articles are both pro-tobacco and anti-tobacco and focus on cigarettes^47,130^. Some articles appear in multiple rows so totals are more than 144. a References^9,10,13-19,21-25,30,32,35-38,40-42,47,48,73,77,78,87-92,103,104,113,114,116,126,129,130,136-150^ b References^47,50-59,61-68,70-72,79-85,97-102,109-112,118,120-122,127,128,130,132,151-158^ c References^13,29,37,43,44,104,159,160^ d References^46,95,104-107,119^ e References^31,34,45,104,145,161-163^ f Reference^95^ g Reference^164^ h References^20,78,150^ i References^69,165^ j References^12, 20,26-28,33,38-39,49,74-76,78, 86,104, 115,117,123-125,131^ k References^60,69,108^		

Table 3 displays the 144 articles by vulnerable population. Articles were listed in multiple categories if more than one vulnerable sociodemographic group was included (e.g. lower SES women). The most prevalent category was race/ethnicity with 35% (n=51) pro-tobacco and 21% (n=30) anti-tobacco.

**Table 3:** Articles by tobacco focus and vulnerable population group

*Population group*	*Pro-tobacco marketing*	*Anti-tobacco campaigns*
Women of reproductive age	14^a^	7^b^
Pregnant women	2^c^	4^d^
Race and ethnicity	50^e^	30^f^
African American	25	9
Hispanic/Latino	7	6
Asian/Pacific Islander	4	1
American Indian/Alaska	1	2
Native	17	13
Multiple		
Sexual minorities – LGBT	4^g^	3^h^
Low socioeconomic status	21^i^	15^j^
Urban and rural areas	15^k^	6^l^
Military/veterans	6^m^	4^n^
Mental health disorders	3^o^	2^p^
Medical co-morbidities	1^q^	0
Total	91	55
Total articles N=144. Two articles are both pro-tobacco and anti-tobacco^47,130^. Some articles appear in multiple rows so totals are more than 144. LGBT: Lesbian Gay Bisexual Transgender a References^15,44,73,86-92,95,137,144,145^ b References^50,97-102^ c References^140,162^ d References^102,152,153,157^ e References^9,10,12-49,136,137,139,141,150,159-161,163,166^ : African American^9,10,12-17,20-25,27-31,43-45,49,147,159^ ; Hispanic/Latino^24,27,32-35,141^ ; Asian/Pacific Islander^33,36,37,48^ ; American Indian/Alaska Native^38^ ; Multiple Groups^18,19,26,39-42,46,47,136,137,139,150,160,161,163,167^ f References^47,50-72,97,100,151,157,158,165^ : African American^61,62,66-69,72,97,151^ ; Hispanic/Latino^59,60,66,70,157,158^ ; Asian/Pacific Islander^165^ ; American Indian/Alaska Native^50,64^ ; Multiple Groups^50-58,63,65,71,72^ g References^123-126^ h References^71,127,128^ i References^18,19,26-29,39,40,42,45,73-78,86,95,143,146,148^ j References^57,63,65,67,79-85,102,154-156^ k References^9,13,28,39,43,74,95,103-107,138,142,160^ l References^50,108-112^ m References^113-117,119^ n References^118,120-122^ o References^129-131^ p References^130,132^ q Reference^129^		

### Race and ethnicity

#### Pro-tobacco marketing

One-third of the articles (35%, n=51) examined pro-tobacco marketing to racial and ethnic minority populations. Most focused on AA, and far less on HL, API, multi-ethnic, ‘minority’ or ‘non-White’, populations. Surprisingly, almost no studies address tobacco marketing involving AI/AN populations, which have the highest smoking prevalence in the US^2,6,7^.

##### African Americans

Several analyses describe the tobacco industry’s history of targeting AAs with menthol cigarette marketing and donations to AA leadership organizations to improve its reputation in these communities^9-14^. Menthol cigarettes were marketed to AAs using culturally targeted messaging and images, implying potential healthful effects of menthol, building on cultural perceptions of mint as medicinal, and creating stronger menthol-flavoured cigarettes appealing to the taste preference of AA smokers^9,10,15,16^. Specific brands such as Newport and Kool targeted promotions to the AA community by featuring hip-hop culture and music and by placing menthol ads in AA magazines^9,14,16^. One of these marketing campaigns, Kool MIXX, was found to have violated the Master Settlement Agreement of 1998, which restricted targeted-marketing to youth^16^.

Tobacco advertisements were more prevalent in AA neighbourhoods^17,18^, at stores in AA neighbourhoods^18-21^, near schools with more AA students^22^, and in AA newspapers^23^ and magazines^14,24,25^. The density of tobacco retail outlets was higher in AA neighbourhoods than in White neighbourhoods^26,27^, and these stores were more likely to have discount promotions and lower prices for menthol cigarettes^9,22^. In New York and Missouri, tobacco retailer outlets were not only denser in poor and in African American communities, they were also located in close proximity to schools, demonstrating the utility of a potential ban on tobacco sales near schools to lower disparities in tobacco retail density^28^. Retailer density may have increased exposure to tobacco promotions. A national sample of retailers found more than twice the odds of price promotions and sale of flavoured cigars in AA neighbourhoods^29^. AA youth living near tobacco outlets reported higher intentions to smoke and greater number of days smoked^30^.

Multiple studies among AAs showed associations between pro-tobacco marketing exposure and tobacco use^12,25,30,31^. For example, pro-tobacco advertisement exposure was positively associated with purchasing cigarettes and smoking more cigarettes among AA smokers^12^. In a study of hospitalized smokers, White smokers were almost twice as likely to report exposure to e-cigarette ads (mostly through stores and the Internet) compared to AA smokers (mostly through radio and television); however, ad exposure was associated with e-cigarette use among AA smokers but not among White smokers^31^. In a more promising direction, one study showed that a decline in cigarette print advertising featuring AA models in AA magazines was associated with a decline in smoking initiation among AAs^25^.

##### Hispanics/Latinos

Targeting of HLs by the tobacco industry was based on segmentation by English/Spanish language comfort, acculturation, country of origin, and geographical region in the US^32,33^. Similar to strategies with AAs, research of industry documents indicated R. J. Reynolds Tobacco sponsored live music festivals with HLs, developed ties to HL leadership organizations like the US Hispanic Chamber of Commerce^32^ and advertised in specific HL communities such as along the US–Mexico border^33^.

Evidence of targeted marketing to HLs usually aggregated groups by language and general HL ethnicity rather than country of origin. Language-based marketing appeared to be effective among HLs; e-cigarette usage was higher among English speakers with greater access to English language marketing, versus non-English speakers^34^. Some studies documented greater tobacco retailer density in HL compared to White neighbourhoods^26-27^ but other studies did not find differences for store advertising by neighbourhood^18^ or for Spanish-language magazines relative to English-language magazines^24^. Menthol was also a marketing feature; one study found that tobacco ads published in Spanish-language women’s magazines were more likely to be for menthol brands relative to ads in English-language women’s magazines^35^.

##### Asians/Pacific Islanders

Research on advertising to API populations was limited and usually aggregated all API populations into one group. Industry documents revealed plans to market menthol cigarettes to Asian Americans because menthol cigarettes were popular among young women in several Asian countries of origin^15,33^. Industry targeting of API populations in the US focused on promoting shared values of cultural identity through smoking, including collectivism and hybrid Asian/Western values^36^. Philip Morris trained store distributors in cultural sensitivity and developed retail-marketing materials for Asian American store clientele^36^. Retail marketing surveillance in California revealed that the cheapest cigarettes cost even less in neighborhoods with a higher proportion of API populations^37^. Studies on the association of marketing with tobacco use were limited for these populations. Among Asian American youth, receptivity to tobacco marketing was associated with regular smoking^31^.

##### American Indians and Alaska Natives

No study was identified on pro-tobacco marketing specifically directed toward AI/AN populations. One study analyzed ways in which the tobacco industry used AI imagery (e.g. the Natural American Spirit headdress), and marketed tobacco as ‘natural’ and ‘traditional’^38^. However, this study was about the use of AI imagery rather than about marketing directed at this population.

##### Multi-ethnic

Several studies examined tobacco retailer density related to neighbourhood ethnicity and income. Density of retailers selling tobacco was positively associated with the proportion of AA residents, negatively associated with the proportion of API residents, and not associated with HL residents^39^. Greater tobacco outlet density was found in areas categorized generally as predominantly non-White or minority populations that either included multiple groups or did not define ethnicity^40,41^. This density link may have been related, in part, to income. One study found more outdoor tobacco advertisements in neighbourhoods with larger proportions of non-Whites, which also corresponded with lower income areas^40^. In that study, the finding may have been due to laws restricting exterior signage in higher income areas^40^. Studies that examined advertising and promotions within stores found differences in communities with more multi-ethnic populations. For example, another study found higher prices of discount and premium cigarettes in stores in areas with more minority residents, although there were no differences in menthol prices^41^. More African American and HL smokers and more lower-income smokers reported exposure to in-store tobacco marketing compared to White Non-Hispanic and higher income smokers in Nebraska^42^.

Few studies have focused on racially and ethnically targeted pro-tobacco marketing of products other than cigarettes, as described in Table 3. Little cigar or cigarillo advertisements were more prevalent in stores in AA neighbourhoods^13,43^. The price of a single Swisher Sweet cigarillo was significantly lower in AA neighbourhoods than in White neighbourhoods^37^. Tobacco companies marketed menthol little cigars to AAs by featuring hip-hop culture and music^44^. Two cross-sectional studies examined exposure to e-cigarette marketing among AAs^45^. AA neighbourhoods had more e-cigarette advertising on store exteriors^45^. One study found that AAs reported more e-cigarette advertising exposure from radio or television than Whites, and e-cigarette advertising was associated with e-cigarette use among AAs^31^. One study of retail outlets found that smokeless-tobacco (ST) advertisements were most prevalent in AA and Asian neighbourhoods^46^.

Only two studies of associations between pro-tobacco marketing exposure and tobacco use explicitly examined racial/ethnic variation in the strength of those associations^30^. One study found that smoking on TV and pro-tobacco advertisements in stores were associated with adolescent smoking susceptibility but the strength of this relationship did not differ by race/ethnicity^30,31^. The other study found that e-cigarette advertisement exposure was associated with e-cigarette use among AAs but not among Whites^31^.

Comparisons across studies were difficult because most studies focused on one type of marketing and one population or community. Measures of pro-tobacco marketing exposure among respondents varied widely, from self-reported recall to media receptivity measures such as having a favourite brand or willingness to use a tobacco promotional item. Despite these methodological issues, most of these studies consistently found positive associations between tobacco marketing exposure/receptivity and smoking behaviour^47-49^.

#### Anti-tobacco campaigns

About one-fifth (21%, n=30) of the articles focused on anti-tobacco campaigns for racial/ethnic minority populations. Most population-based studies of awareness and effectiveness of anti-tobacco campaigns found similarities, rather than differences, across racial/ethnic groups^50-54^. For example, exposure to the ‘truth’ campaign, a major national youth prevention campaign, was associated with lower risk of smoking initiation, lower intentions to smoke in the next year, and more negative attitudes about tobacco companies across racial/ethnic groups^53,55^. Across state anti-tobacco campaigns, higher campaign exposure was associated with decreased odds of smoking across racial/ethnic groups^54^.

However, some anti-tobacco media campaigns showed differential effects across racial/ethnic groups. For example, awareness of the ‘EX’ mass media campaign was associated with quit attempts among AA adult smokers, but not among White or HL smokers^56,57^. A multi-channel campaign promoting the 2006 Nicotine Patch Program had similar effects across racial/ethnic groups in raising awareness, but HL and AA adult smokers were more interested in the program than were White and API smokers^58^.

There was limited research on differential responses to anti-tobacco message themes and modalities^59-62^. Graphic and emotional anti-smoking ads in one study were associated with quit attempts among AA and White smokers, but not among HL smokers^63^. A study among AI/ANs found reactions of anger, sadness and worry to graphic warning labels depicting children but did not compare reactions among other populations^64^. Another study found that non-White participants were more likely to respond to online ads for cessation treatments than to traditional media^65^. A general media campaign promoted more calls to the Massachusetts quitline among White smokers, while targeted provider outreach was more effective at increasing quitline referral rates among AAs and HLs^66^. These differences identified a need to target outreach efforts and message design to specific audiences but were limited in comparing groups.

Culturally targeted messages incorporating cultural values and beliefs were explored in several studies. Messages based on cultural beliefs about smoking among low-income African American smokers were more likely to contribute to intention to quit compared to non-targeted messages^67,68^. A social media-based video campaign for Somali American youth used youth-driven messaging focusing on social and religious norms about tobacco use^69^. Messages, framing and channels of delivery were assessed for Spanish-speaking smokers to guide them to an online cessation program (*Become an Ex*)^70^; viewers were more likely to click on website banners on the Spanish version of Yahoo compared to other websites, and on banner ads with themes of loss-frame over gain-frame, familism rather than fatalism, and ads targeted to characteristics such as language and dress compared to other ads. In a study that compared the ethnicity of viewers with the intended ethnicity of anti-tobacco ads, the viewers liked the ads more if they thought the intended audience matched their own ethnicity^71^.

Perception and recall of anti-smoking advertisements also differed by race/ethnicity. HL compared to White adolescents reported less exposure to anti-tobacco advertisements at schools and sporting events^72^. AA adolescents recalled fewer television and poster anti-smoking advertisements than White adolescents but recalled more ads at movies and live sporting events.

### Low socioeconomic status (SES)

#### Pro-tobacco marketin

Studies of tobacco marketing to low SES populations represented 15% (n=22) of the articles. Most of these studies analyzed tobacco industry documents or described point-of-sale marketing in low-SES neighbourhoods.

Tobacco companies historically identified ‘working class,’ ‘less-educated’ and ‘present-oriented’ consumers as an important market^73^. For instance, an R.J. Reynolds project in 1976 distributed cigarette coupons with food stamps to ‘welfare mothers’. Initially, tobacco companies developed cigarette brands specifically for lower SES consumers, but the approach shifted over time to include these demographics in marketing for established brands (e.g. Marlboro)^73^.

Higher tobacco retailer density^19,27^, lower tobacco prices^37^, and more tobacco marketing^19,40,74-76^ were found in lower SES than in higher SES communities. Stores in lower income neighbourhoods also had more ads for menthol cigarettes^18^. It is unclear whether this indicated targeting to racial/ethnic minorities or to low-SES populations, as these vulnerabilities often co-occurred in the same neighbourhoods^19^. Cigarette prices were lower in stores near public schools compared with private schools^77^. Proximity to tobacco retailers was important because smokers living in high poverty areas close to tobacco retailers were less likely to quit smoking and had lower processation attitudes than those living farther away or in higher SES areas^78^.

#### Anti-tobacco campaigns

Studies of anti-tobacco campaigns to low SES populations represented 10% (n=15) of the studies identified. Several examined message design, finding that advertisements using strong negative emotions, testimonials or graphic imagery were more effective in motivating low-SES smokers to quit compared to high-SES smokers^63,79-81^. Viewing a web-based anti-smoking ad changed implicit attitudes toward smoking among smokers with less formal education^82^. However, anti-smoking campaigns that stigmatized smoking had a boomerang effect in which exposure significantly lowered their cessation intentions^37^. A qualitative study on anti-smoking messages found culturally-targeted messages were more appealing to lower income smokers than other messages^67^. However, these low-SES smokers also reported scepticism about cessation messages and barriers to quitting related to stress, social contexts, and addiction^83^. Among blue collar construction workers, smoking cessation ads that emphasized family and work and presented smoking harms in the context of work hazards were more appealing than other messages^84^. Only a few studies examined avenues for reaching lower SES populations. Two of these studies found higher awareness of anti-tobacco campaigns among individuals or neighbourhoods with higher formal education^57,85^. One study that examined channels to reach populations found that online ads promoting cessation treatments reached a higher percentage of smokers with a high school education or less compared to more traditional media channels^65^.

### Women of reproductive age and pregnant women

#### Pro-tobacco marketing

Women of reproductive age (average 18–51 years old) were targeted by tobacco companies, as described in 10% (n=14) of our pro-tobacco articles. They are a vulnerable population because of the complications of tobacco use during pregnancy if they begin using tobacco and then are unable to quit. The companies used multiple methods, including female-focused advertising campaigns, product/package design, and targeting specific subgroups of women. One of the first industry marketing efforts, in the 1920s, marketed Lucky cigarettes as an appetite suppressant^86,87^. In the 1960s, PM launched Virginia Slims, one of the first American female-marketed cigarette brands, with themes about money and materialism^88^. Other female-focused cigarette brands appeared during the 1980–90s^73,86,87,89,90^. Each brand reflected a specific female image or niche: Dakota (young women with blue-collar jobs, street-smarts, and toughness); Virginia Slims (stylish and status conscious women)^73^; and Chelsea (thrifty women who had less formal education)^86^. Tobacco companies also targeted military wives, inner-city minority women, and price-sensitive women^86^. Military wives were perceived to be a captive audience of young women with lower income, less formal education and geographical isolation who could form lifelong brand loyalties and could produce word-of-mouth advertising on the military base^86^. Inner-city AA women were perceived as being of lower income, concerned with present needs, having extended family obligations, and being price-sensitive. To reach this group, R.J. Reynolds reduced pack prices at retail, advertised in locations such as clubs, bus stops, and beauty salons, and distributed free fingernail decals and earrings with Salem logos. Tobacco companies marketed cigarettes as a small indulgence to compensate for personal sacrifices among price-sensitive women^86^.

Analysis of tobacco documents identified product design features that were attractive to women and appealed to their social and health concerns, including small, colourful packs, slim cigarettes, lower tobacco content, milder tobacco, slower burn rate, flavours, and low side-stream emissions to decrease secondhand smoke^89^. Women also preferred cigarette packs with overtly female designs and flavours, and associated them with popularity, attractiveness, slimness, glamour, and lower health risks^87^.

Several studies assessed the reactions of adolescent and adult women to tobacco advertisements with various images and themes. The advertising campaign for Camel No. 9, which was launched in 2007, was followed by a 10% rise in adolescent girls who nominated a favourite cigarette advertisement, compared to earlier years. Nomination of a favourite ad was a significant predictor of tobacco experimentation in this population^90^. Adolescent girls also rated female-valenced ads^91^ and ads with relaxation themes^92^ more highly than other ads, perhaps because these ads generated self-relevance and positive affect. Female-focused marketing also used themes of thinness and popular celebrity usage^89,93,94^.

Although most tobacco marketing research focused on cigarettes, research on tobacco industry documents indicated that the launch of snus in the 2000s was partially an effort to attract urban women to ST^95^. Emerging products such as e-cigarettes, snus, and dissolvables were identified in documents research as potentially appealing to women^95,96^.

#### Anti-tobacco campaigns

Anti-smoking media campaigns for women of reproductive age comprised 5% (n=7) of the articles; and 3% (n=4) focused on pregnant women, usually reporting effects of specific campaigns. For example, ‘One Tiny Reason to Quit’ ran in 2009 and 2011, targeting pregnant AA women with gain-frame messages that offered benefits of quitting^97^. The campaign was disseminated via multiple channels in high-risk neighbourhoods, community venues and AA media outlets. During the campaign, the proportion of pregnant AA women who called the quitline increased.

Several types of anti-tobacco messages were effective among women. Both high-fear (e.g. risk of death or tracheotomy) and low-fear arousal messages (e.g. tobacco industry is deceptive, smokers appear foolish) resulted in lower intentions to smoke, more negative attitudes toward smoking, greater susceptibility to anti-smoking ads, and more negative beliefs about the acceptability of smoking among women^98^. Empathy appeals (campaigns that represented someone’s pain, interpersonal relationships, or emotions) were more effective for women than for men, but fear appeals were equally effective for both sexes^99^. Ads that emphasize short-term consequences of smoking (e.g. social rejection, appearance) were more effective at reducing smoking behaviour and increasing intentions to quit among women, but messages with long-term consequences (e.g. disease, death) were more effective at reducing intentions to start smoking in women^100^. Online ads about health effects of smoking were more effective among women than ads that empowered viewers or suggested ways to quit smoking^101^. Among low-SES smoking mothers, health messages about protecting children from secondhand smoke were associated with intentions to quit when the source of the message was personal (i.e. friends, family) or a physician, while health messages from dentists were associated with lower child tobacco smoking exposure^102^.

### Urban/rural areas

#### Pro-tobacco marketing

Geographically defined communities considered vulnerable to tobacco industry marketing included inner city urban and rural communities, analyzed in 10% (n=15) of the studies. The tobacco industry identified urban ‘focus’ communities that were predominantly low-income and AAs with high menthol sales as an important part of their strategy to recover declining cigarette sales in the US^9^. Marketing practices included contracts with these retailers to ensure prominent product displays, cigarette packaging appealing to smokers, discount programs to increase retailer profits in exchange for control over how stores offer products, and urban life-oriented campaigns to increase menthol cigarette sales. Urban census tracts also were more likely to have tobacco ads within 500 feet of schools, playgrounds and churches, in violation of the Master Settlement Agreement^74^.

Rural youth reported more cigarette smoking and more exposure to retail tobacco advertising than urban youth^103^. However, sociodemographic factors, cigarette taxes, and tobacco ad exposure did not entirely explain urban/rural disparities^104^. A qualitative study of smokeless tobacco use among rural male youth found that product characteristics (i.e. brands, flavors, and packaging) encouraged continued ST use, while availability of flavors and seasonal offerings encouraged experimentation^105^.

Rural areas were targeted by smokeless tobacco (ST) marketing aimed at low-SES men. Industry documents showed that R.J. Reynolds reached rural, low-income males through ST sampling, television commercials and sponsorship at fishing, rodeo, and baseball, events^95^. Qualitative research suggested that marketers of ST capitalized on perceptions of masculinity in rural communities to reinforce initiation and continued ST use^106^. Focus group and interview participants in Appalachian Ohio confirmed the ease of obtaining ST and how packaging and advertisements reflected male cultural standards of their communities^106^. After the Family Smoking Prevention and Tobacco Control Act of 2009 was enacted, there was a reduction in the frequency of tobacco ads at retail outlets. However, the proportion of stores advertising ST did not significantly change and the number of ST brands being advertised doubled between baseline and follow-up^107^.

#### Anti-tobacco campaigns

We identified anti-tobacco campaigns toward urban or rural areas in 4% (n=6) of the studies. Rural youth recalled and perceived ads differently from more urban populations. Rural youth in Indiana were less likely to recall anti-tobacco media messages than suburban adolescents^108^. Nevertheless, anti-tobacco media campaigns were able to reach rural communities. The national ‘truth’ campaign expanded to reach rural and low-population-density area youth by purchasing local broadcast media. Confirmed awareness increased from 40% to about 70% among youth in those rural media markets^109^. Rural youth were highly receptive to ‘truth’ advertisements, though never smokers were more receptive than ever smokers^109^. In ‘The Plain Truth’ campaign, TV and radio ads depicting graphic health harms from tobacco were highly recalled and perceived as effective by both American Indian and White youth in the Northern Plains region of the US^50^. Response to ads may have differed by smoking status of the youth. Rural high school students who used tobacco were more likely to perceive the national ‘truth’ campaign as ineffective and to hold negative perceptions of anti-tobacco messages compared to non-users^110^.

Among rural adults, local media, technology, billboards, and print, were considered more effective than state-wide media channels to promote secondhand smoke and smoke-free policies^111^. Print media were an important source of exposure to anti-tobacco campaigns in rural populations. Print messages that contained negative emotional tone, loss framing, appeals to religiosity and shifting focus away from smokers were perceived as effective strategies for promoting support for smoke-free policies in rural communities^112^.

### Military or veterans

#### Pro-tobacco marketing

A few studies (4%, n=6) documented the pervasiveness of pro-tobacco promotions in military life. Soldiers reading military newspapers were exposed to an over-representation of pro-tobacco content and an under-representation of tobacco control messages^113^. Tobacco advertising in *The Military Times* magazine, widely read by military personnel, had no cigarette or other combustible tobacco ads but frequently contained ST ads. On military bases, tobacco was sold at substantially lower prices than at public retail outlets^114^.

In the 1980–90s industry documents identified 1400 tobacco-sponsored events for military personnel in the US and abroad, until this form of event marketing was restricted by the Master Settlement Agreement^115^. These market plans revealed strategic efforts to increase tobacco sales volume among military personnel by selling through military outlets and attracting young men of a specific lifestyle and SES who could carry tobacco use into civilian life^116^. Marketing plans included in-store merchandising, event sponsorship, development of brands appealing to military personnel, and legislation protecting these tobacco promotions. A case study of the Gulf War (1990–91) demonstrated use of industry-sponsored free samples, direct mail, functional items with brand names on them (e.g. an item of clothing with a cigarette brand name on it), and tobacco-sponsored events that assisted communication with families and welcomed troops home. As a result, tobacco companies were perceived as benefactors, often receiving positive support from military authorities^117^.

#### Anti-tobacco campaigns

Smoking was more prevalent among enlisted junior personnel than the general population, and nearly half reported that they began smoking after enlisting^118^. However, few studies analyzed efforts to reduce tobacco use in this population (3%, n=4). Anti-tobacco articles received limited coverage in military magazines compared to other health topics^119^. A focus group study of Air Force and Army personnel identified themes that could deter smoking in this population: smoking can lead to early discharge, lessen the ability to fight, reduce productivity, and ability to lead others. However, messages typically used with young adults about tobacco industry manipulation and health effects had less support^118^. A different focus group study of US military personnel examined myths about smoking and found that all participants believed that tobacco served military needs by reducing stress, fitting in with others and helping them take breaks, despite known effects of tobacco on fitness^120^. Media coverage of the hazards of secondhand smoke helped pass a smoking ban in Navy submarines^121^. Among Air Force trainees^122^ exposure to existing anti-tobacco advertisements developed for the general population led to increased perceived harm and reduced intentions to use tobacco products. Among these ads, those that portrayed the negative effects of tobacco on health or sexual performance and revealed tobacco industry manipulations were most effective.

### Sexual minorities (LGBT)

#### Pro-tobacco marketing

Only 3% (n=4) of the articles focused on pro-tobacco marketing to LBGT populations; with most drawn from documents research of industry marketing practices. Philip Morris first placed ads in a national LGBT magazine in 1992. Since then tobacco advertising in weekly LGBT newspapers and magazines is fairly common^123^. Most ads featured sexual ambiguity rather than overtly LGBT individuals^123,124^. For example, Philip Morris created ads with a man and woman when targeting the general public, but added a second man or woman to the ad when targeting the LGBT community^123^. In addition to tobacco ads (typically large and image-based) and smoking cessation ads (typically small and text-based), LGBT periodicals also contained numerous images of celebrities smoking^124^.

Tobacco companies also attempted to gain loyalty from the LGBT community and improve their corporate image by: donating to HIV/AIDS causes; publicizing their anti-discrimination, anti-harassment, and diversity-awareness business policies^123^; sponsoring performing arts; and giving away tobacco samples and coupons at events in LGBT communities^125^. In a focus group study, LGBT individuals perceived the tobacco industry targeting as a form of social acceptance and an opportunity to increase their visibility to the general population^126^.

#### Anti-tobacco campaigns

Only 2% of the studies focused on anti-tobacco campaigns for LGBT populations. Awareness of ads promoting smoking cessation was similar among LGBT individuals and their heterosexual counterparts^127^. In a study that compared reactions to anti-tobacco ads targeting AA, HL and LGBT populations, support for anti-tobacco messages was lowest for the LGBT themed ads but higher if someone self-identified as LGBT compared to those who did not^71^. A 2013–14 anti-smoking educational campaign (*Break Up*) for LGBT individuals in Los Angeles County, featured graphic advertisements online and in bars, clubs, and gyms in areas of the county with the highest concentration of businesses that service the LGBT community. Approximately one-third of LGBT participants were aware of the campaign. Among those aware of it, more than one-third had discussed the campaign with someone else, while one-fourth shared the campaign on social media. Awareness of the campaign among LGBT smokers was associated with seriously thinking about quitting and ever having taken steps to quit smoking; however, awareness of the campaign was not associated with smoking cessation^128^.

### Mental health disorders

#### Pro-tobacco marketing

Only 2% of the articles focused on pro-tobacco marketing to people with mental illness. Tobacco industry documents revealed strategies for marketing cigarettes to people who were homeless or had serious mental illness, and industry alliances with providers of services to these populations^129^. Smoking among patients in substance-use treatment was associated with high exposure and receptivity to tobacco advertisements^130^. A San Francisco study also found higher exposure, with a twofold greater tobacco retailer density in neighbourhoods of smokers with severe mental illness compared to the general population^131^. In that study, greater retail availability was associated with poorer mental health, greater nicotine dependence and lower self-efficacy for quitting among residents with severe mental illness.

#### Anti-tobacco campaigns

We identified only one article examining smoking-related health messaging among people with mental illness^132^. That study found that young people with psychotic disorders responded favourably to both picture and video health warnings, and that such media messages can be highly effective among people with psychosis.

## DISCUSSION

This review highlighted important gaps in the research on pro-tobacco marketing and anti-tobacco campaigns for vulnerable populations in the US. These gaps included certain types of tobacco products, at-risk populations, and corresponding marketing strategies.

Almost all of the pro-tobacco marketing literature focused on cigarettes or more generally on ‘tobacco’ as a general product, followed in frequency by cigars/little cigars and e-cigarettes. One pro-tobacco study was found on hookah/waterpipe, one on snus and none on pipe or roll-your-own tobacco. In the past five years some analyses have emerged for e-cigarettes. No tobacco prevention or cessation campaign was identified on cigars/little cigars, e-cigarettes, smokeless tobacco, snus, pipe or roll-your-own tobacco. These gaps may reflect the market share of cigarettes but provides little evidence to inform campaigns to prevent use of emerging tobacco products.

There were also notable gaps for certain populations. Most studies on pro-tobacco marketing involved women of reproductive age, AAs, people with low SES, and inner city urban or rural populations. Among these studies, several addressed the intersections of vulnerable groups defined by race, SES, and urban neighbourhood. However, there were very few studies on pro-tobacco marketing aimed at sexual minorities, AI/ANs and people with medical co-morbidities or mental illness. These populations could be analyzed using tobacco industry documents and more current survey research. For example, a recent analysis of industry documents revealed that tobacco companies targeted AI/AN populations with price reductions, coupons, giveaways, charitable sponsorships, and non-evidence-based youth smoking prevention programs, as well as capitalizing on the sovereign status of Tribal lands, stores, and casinos, to sell cigarettes tax-free^133^. In the anti-tobacco literature on tobacco prevention and cessation campaigns, there were major population gaps for pregnant women, LGBT populations, APIs, AI/ANs, military/veterans, and/or those with mental health or other medical co-morbidities, despite the high tobacco related risks for these populations. The evidence for tailored messages and targeted channels for these specific high-risk populations would be useful but it is sparse. Additional empirical research on these populations is recommended since such data may be helpful for understanding disparities in tobacco use and developing more effective tobacco control strategies.

The studies on pro-tobacco marketing strategies documented common industry tactics that segmented consumers and optimized appeal and uptake of tobacco products. These tactics included designing products, packaging and advertisements to appeal to population niches, reducing prices, advertising in sociodemographically targeted outlets and locations, and donating to leadership organizations to gain loyalty from vulnerable communities. Research on the methods of targeted anti-tobacco campaigns found that specific messages tailored for subgroups, when used, appeared to be more salient and acceptable to vulnerable population groups. However, broad-reach campaigns were also found to be effective in reaching across subpopulations when combined with strategic placement in population-specific channels and media. Additional research is needed to understand geographical and SES variations that could improve access to and understanding of campaign messages as well as the impacts, reach and limitations of general campaigns, compared to targeted campaigns.

Well-designed mass marketing prevention and cessation campaigns using culturally appropriate media, languages, channels and message designs are needed in combination with tobacco control policies to counter pro-tobacco marketing and reduce tobacco use disparities^134^. The passage of the Family Smoking Prevention and Tobacco Control Act in 2009 granting the Food and Drug Administration the authority to regulate tobacco products provided new opportunities for a combination of communication campaigns and regulations to prevent the harms of tobacco product use in vulnerable populations. For example, in 2015, the Food and Drug Administration launched *‘Fresh Empire’*, a national campaign for at-risk multicultural youth who identified with hip-hop culture, specifically AA, HL and API youth. In 2016, it launched *‘This Free Life’*, a public education campaign to prevent and reduce tobacco use among LGBT young adults. These campaigns are taking place at the same time as evolving federal regulatory efforts to improve warning labels and to limit flavours (other than menthol and tobacco flavours) and e-cigarettes. Existing federal regulations limit claims of ‘light’, ‘mild’, and ‘low-tar’ products, sales to youth, tobacco brand sponsorship, branding of functional items and services, and free samples. State regulations and legal settlements have provided additional restrictions on tobacco marketing not yet found at the federal level.

Additional tobacco control policies suggested by our literature review can help lessen the impact of tobacco on vulnerable groups. For example, tobacco signage, displays, discounts and sales in retail outlets can be limited to shelving separated from public view, to reduce exposure to non-tobacco customers. The geographically targeted retailer density and point-of-sale marketing of tobacco products to vulnerable communities could be further limited with retail licensing that charges licensing fees that reduce the number of stores selling tobacco and restrict the location or visibility in proximity to schools, parks, and mental hospitals^135^. Tobacco advertising in magazines can reach very specific populations of tobacco users and non-users with persuasive characteristics that resonate with the viewers. A restriction on print advertising would limit this type of targeted effort. Tobacco advertising on Internet sites also has the potential to reach both broad and targeted audiences regardless of age or tobacco use status. This reach could be diminished with advertisements limited to sites that use gateways that stop viewers unless they can demonstrate that they are at least 21 years of age and use tobacco. Finally, requiring plain packaging also could prevent the tobacco industry from using design features that attract vulnerable populations.

Although this literature review focused on findings from research conducted in the United States, similar anti-tobacco and pro-tobacco strategies, most likely, are being used in other countries and continue to impact smoking prevalence among vulnerable populations. The gaps and challenges identified here can provide insights into potential areas of focus for countries working under the Framework Convention on Tobacco Control worldwide (which the US has not yet ratified) and with high risk populations defined by minority, geographical, SES, military and medical status.

### Strengths and limitations

The types of research studies included qualitative studies, observational studies identifying patterns of exposure, awareness, and attitudes at the population level, point-of-sale marketing studies, documents research revealing industry marketing strategies, and laboratory studies comparing media perceptions and effects. We may have missed literature not covered in our data bases, such as advertising studies from predominantly business databases. The nature of our search process yielded results that made comparisons difficult across populations or types of campaigns, and limited our ability to address how targeted marketing affects vulnerable populations differentially. At the same time, this heterogeneity revealed a wide array of marketing practices that informed this analysis. Many studies lacked detail that could lead to counter-marketing recommendations due to aggregating groups like API or LGBT. Research on retail outlets did not always specify the SES or racial/ethnic make-up of local communities. Comparable measures and sampling designs would facilitate comparisons between studies in the future. Finally, few data existed on the use of new marketing channels of mobile and social media platforms for targeting vulnerable groups.

## CONCLUSIONS

This study provides an extensive review of literature from multiple disciplines involved in marketing research. It identifies several patterns of pro-tobacco marketing or anti-tobacco campaigns that can be used to design regulatory and communication strategies to prevent smoking, improve cessation, and reduce health disparities. However, there are serious gaps in research on marketing affecting some of the most vulnerable populations. Most notably, there is a need for studies on anti-tobacco campaign strategies reaching these groups. These gaps need to take priority in future planning efforts at the local, state, and national level.

## CONFLICTS OF INTEREST

The authors have completed and submitted the ICMJE Form for Disclosure of Potential Conflicts of Interest and none was reported.
